# Differential Expression of the *TLR4* Gene in Pan-Cancer and Its Related Mechanism

**DOI:** 10.3389/fcell.2021.700661

**Published:** 2021-09-23

**Authors:** Jialing Hu, Jiasheng Xu, Xiaojin Feng, Yiran Li, Fuzhou Hua, Guohai Xu

**Affiliations:** ^1^Department of Anesthesiology, The Second Affiliated Hospital of Nanchang University, Nanchang, China; ^2^Department of Surgical Oncology, Zhejiang University Cancer Center, Hangzhou, China; ^3^Queen Mary College, Nanchang University, Nanchang, China

**Keywords:** immune cell infiltration, toll-like receptor 4, pan-cancer, prognosis, bioinformatics

## Abstract

Previous studies have revealed the relationship between toll-like receptor 4 (*TLR4*) polymorphisms and cancer susceptibility. However, the relationship between *TLR4* and prognosis and immune cell infiltration in pan-cancer patients is still unclear. Through the Genotype-Tissue Expression (GTEx) and The Cancer Genome Atlas (TCGA) databases, the distinct expression of the *TLR4* gene in 24 tumors and normal tissues was analyzed. Univariate Cox proportional hazards regression analysis was used to identify the cancer types whose *TLR4* gene expression was related to prognosis. The relationship between *TLR4* and tumor cell immune invasion was studied. Spearman’s rank correlation coefficient was used to analyze the relationship among *TLR4* and immune neoantigens, tumor mutation burden (TMB), microsatellite instability (MSI), DNA repair genes, and DNA methylation. Gene Set Enrichment Analysis (GSEA) was used to identify the tumor-related pathways that the *TLR4* gene was highly expressed in; the expression of the *TLR4* gene was verified with the Human Protein Atlas (HPA) database. Low expression of *TLR4* was associated with an inferior prognosis in kidney renal clear cell carcinoma (KIRC), skin cutaneous melanoma (SKCM), and uterine corpus endometrial carcinoma (UCEC), while high expression was related to a poor prognosis in head and neck squamous cell carcinoma (HNSC), prostate adenocarcinoma (PRAD), stomach adenocarcinoma (STAD), and testicular germ cell tumor (TGCT). The expression of *TLR4* was negatively correlated with the expression of B cells in STAD. The expression of *TLR4* was positively correlated with the infiltration of B cells, CD4 and CD8 T cells, neutrophils, macrophages, and dendritic cells in STAD, KIRC, UCEC, TGCT, and SKCM. The expression of the *TLR4* gene in KIRC, SKCM, STAD, TGCT, and UCEC was highly correlated with inducible T-cell costimulator (*ICOS*), cytotoxic T lymphocyte-associated molecule 4 (*CTLA4*), and *CD28* immune checkpoints. Spearman’s rank correlation coefficient showed that the expression of *TLR4* gene was significantly correlated with TMB in STAD and UCEC and was prominently correlated with MSI in TGCT, STAD, and SKCM. The expression of the *TLR4* gene was highly correlated with MLH1, MSH2, and MSH6 in KIRC, SKCM, and STAD. The expression of the *TLR4* gene was remarkably correlated with the methyltransferases DNA methyltransferase 2 (DNMT2) and DNA methyltransferase 3-beta (DNMT3B) in SKCM and STAD. Enrichment analysis showed that *TLR4* was highly expressed in the chemokine signaling pathway and the cell adhesion molecule and cytokine receptor interaction pathway. In summary, the expression of *TLR4* is linked to the prognosis of KIRC, SKCM, STAD, TGCT, and UCEC patients and the level of immune infiltration of CD4, CD8 T cells, macrophages, neutrophils, and dendritic cells.

## Introduction

Cancer is the second leading cause of death in the world. The rising cancer incidence and death rate and their impact on public health have attracted wide notice, and a great deal of research has been done on the occurrence, development, and metastasis mechanisms of cancer (Torre et al., 2015). Although targeted therapies and extensive treatments for certain cancers have made great progress, a large number of cancer patients still have a poor prognosis. Therefore, there is an urgent need for a useful biomarker that can forecast the prognosis of cancer. Chronic inflammation is closely linked to tumors (Balkwill et al., 2005). It has been discovered that under chronic inflammatory conditions, various mechanisms can promote the occurrence and development of cancer, including activating angiogenesis, inhibiting apoptosis, stimulating cell proliferation, and destroying antitumor immune responses (Baniyash, 2006; Mantovani et al., 2008), and inducing epigenetic changes. It is closely related to the development of cancer.

Previous studies have shown that inflammation-induced Toll-like receptors (TLRs) play a critical role in tumorigenesis and development (El-Omar et al., 2008; Grimm et al., 2010). TLRs play a vital role in innate immunity and activate and mediate the inflammatory response by identifying invading pathogens. Since TLR plays a pivotal role in innate immunity and chronic inflammation is identified as one of the important events in the carcinogenic process, the expression profile of TLR genes may also be a useful marker of early cancer susceptibility, development, and progression. To date, 11 TLR genes (TLR1–11) have been discovered in humans (Takeda and Akira, 2015). **Toll-like receptor 4 (*TLR4*)** is mainly expressed on immune cells, namely, T cells, natural killer cells, dendritic cells, macrophages, and neutrophils (McGettrick and O’Neill, 2007). A previous study also showed that *TLR4* was expressed in the tumor microenvironment, especially in cancer cells (Cammarota et al., 2010; Li et al., 2017). In view of the limited reports of *TLR4* expression in different cancer types, it is challenging to reach any conclusion at present. Comprehensive analysis of *TLR4* expression is necessary; moreover, exploring effective prognostic biomarkers can help optimize the prognostic evaluation system of cancer.

Here, we used specific data to show that the *TLR4* gene is a biomarker for a poor prognosis of kidney renal clear cell carcinoma (KIRC), skin cutaneous melanoma (SKCM), stomach adenocarcinoma (STAD), testicular germ cell tumors (TGCTs), and uterine corpus endometrial carcinoma (UCEC) and to explore the potential signaling pathways that the *TLR4* gene plays in tumorigenesis and development. Compared with the existing *TLR4* gene-related experiments, our work comprehensively and systematically studied the expression of the *TLR4* gene in pan-cancer and screened out cancer types with a poor prognosis, which will provide reference data for follow-up studies.

## Materials and Methods

### Data Acquisition

First, we used the Genotype-Tissue Expression (GTEx) data set [https://commonfund.nih.gov/GTEx/. This database collects data from normal human tissues for sequencing and can be used to study the differential gene expression between different tissues and between normal and diseased tissues. In addition, since The Cancer Genome Atlas (TCGA) mainly collects data from cancer tissues, it can be used in conjunction with TCGA database to ensure more reliable results]. We analyzed the expression level of the *TLR4* gene in 21 normal tissues, in accordance with the tissue source. Second, the data of tumor cell lines were downloaded from the Cancer Cell Line Encyclopedia (CCLE) database (https://portals.broadinstitute.org/ccle, a database of cancer cell lines that currently contains more than 1,000 cell lines), and we analyzed the expression of the *TLR4* gene in each tumor cell line. Then, data were downloaded from TCGA database (https://cancergenome.nih.gov/. TCGA mainly contains data on cancer tissues, including data on 33 types of tumors. The data are comprehensive, covering miRNA, methylation, mutation, and other data). Threshold values were determined according to the following values to analyze the expression differences of the *TLR4* gene in 33 kinds of tumors: ^∗^*p* < 0.05, ^∗∗^*p* < 0.01, and ^∗∗∗^*p* < 0.001. Because of the lack of normal tissue data in TCGA database, we compared the normal tissue data from the GTEx database with tumor data from TCGA database to analyze the differential expression of the *TLR4* gene in 27 types of tumors and adjacent normal tissues.

### TCGA Database Analysis

The prognostic difference of the *TLR4* gene expression group in 33 kinds of tumors was analyzed with the dichotomy method. To explore the types of cancer that are significantly associated with *TLR4* gene expression, we used univariate regression analysis to analyze the prognosis of 33 kinds of tumors, including process-free intervention (PFI), overall survival (OS) rate, disease-specific survival (DSS), and disease-free intervention (DFI). A survival curve was drawn, and the selected cancer types were used for further study and analysis.

### TIMER Database Analysis

We analyzed the correlation between *TLR4* gene expression and six kinds of immune cell scores in 33 kinds of cancer: six kinds of infiltrating immune cells (B cells, CD4+ T cells, CD8+ T cells, neutrophils, macrophages, and dendritic cells) through the tumor immune assessment resource (TIMER) algorithm database^1^. RNA-Seq expression profile data were used to detect the infiltration of immune cells into tumor tissues and provide the infiltration of six types of immune cells (B cells, CD4+ T cells, CD8+ T cells, neutrophils, macrophages, and dendritic cells).

### Relationship Between Toll-Like Receptor 4 Gene Expression and Immunity

To study the relationship between *TLR4* gene expression in KIRC, SKCM, STAD, TGCT, and UCEC and immune checkpoints, we extracted more than 40 immune checkpoints and used Spearman’s rank correlation coefficient to calculate and analyze the correlation between the two (^∗^indicates a mild correlation, *p* < 0.05; ^∗∗^indicates a moderate correlation, *p* < 0.01; ^∗∗∗^indicates a high correlation, *p* < 0.001).

In addition, Spearman’s rank correlation coefficient was applied to analyze the relationship of *TLR4* gene expression and immune neoantigens, tumor mutation burden (TMB), microsatellite instability (MSI), DNA repair genes and DNA methylation, and Gene Set Enrichment Analysis (GSEA) (http://software.broadinstitute.org/gsea/msigdb/index.jsp, an enrichment method proposed by the Broad Institute that categorizes human genes in terms of location, function, etc., to construct many gene sets for scientific research) of the *TLR4* gene in tumors with high and low expression. The Kyoto Encyclopedia of Genes and Genomes (KEGG) database (https://www.kegg.jp. It is a database that integrates genome, chemistry, and system function information and aims to reveal the genetic and chemical blueprints of life phenomena. It can use graphics to show numerous metabolic pathways and the relationships among each pathway) was used to analyze the enrichment of *TLR4* gene expression in the signaling pathways. Finally, to further compare the expression of the *TLR4* gene in the cancer tissues of 14 tumors and the corresponding normal tissues, the Human Protein Atlas (HPA) database^2^ was used. Transcriptomics and proteomics were used as techniques to study protein expression in different human tissues or organs at the RNA and protein levels and to explore the expression of protein-coding genes in normal and tumor tissues or organs. This database was used to verify the expression of the *TLR4* gene in various tumors.

### Immunohistochemical Staining

To further compare the expression of the *TLR4* gene in the cancer tissues of 14 tumors and the corresponding normal tissues, the HPA database was used to verify the expression of the *TLR4* gene in various tumors. To further verify the reliability of the above results, we performed immunohistochemical (IHC) staining. Samples from patients with seven kinds of cancer who were being treated in our hospital were collected [liver hepatocellular carcinoma (LIHC), colon adenocarcinoma (COAD), ovarian serous cystadenocarcinoma (OV), prostate adenocarcinoma (PRAD), breast invasive carcinoma (BRCA), pancreatic adenocarcinoma (PAAD), and UCEC]; three pairs of samples (cancer and normal tissues) were collected for each type of cancer. The samples were embedded in paraffin and incubated with anti-*TLR4* antibody (1:100, sc-293072, Santa Cruz Biotechnology) at 4°C overnight. Then, a secondary antibody coupled to horseradish peroxidase (HRP) (1:500, ab150113; Abcam) was incubated with the sections for 60 min at room temperature, followed by 3,3’-diaminobenzidine (DAB substrate system; Dako) and hematoxylin staining. Images were taken with an Olympus cx-21 (Japan) magnified ×200. Using the analysis software Image-Pro Plus 6.0 (Media Cybernetics, Inc., Rockville, MD, United States), the same intensity of brown color was selected as the unified standard to judge the positivity of all photos, and each photo was analyzed to obtain the positive cumulative optical density (IOD) and tissue pixel area (AREA). The average optical density IOD/AREA (mean density) was calculated. SPSS 19.0 software was used for independent-sample *t*-tests of the average optical density values obtained from the cancer and normal tissues. *p* < 0.05 was defined as statistically significant.

### Statistical Analysis

Single-factor regression analysis was used to detect the expression and prognosis of the *TLR4* gene in different tumors and tissues. Spearman’s correlation coefficients were used to assess the correlation between *TLR4* gene expression and immune infiltration level, immune checkpoint, TMB, MSI, DNA repair genes, and DNA methylation in different types of cancer. A *p*-value < 0.05 was regarded as statistically significant.

## Results

### The Expression Levels of Toll-Like Receptor 4 Gene

**FIGURE 1:**
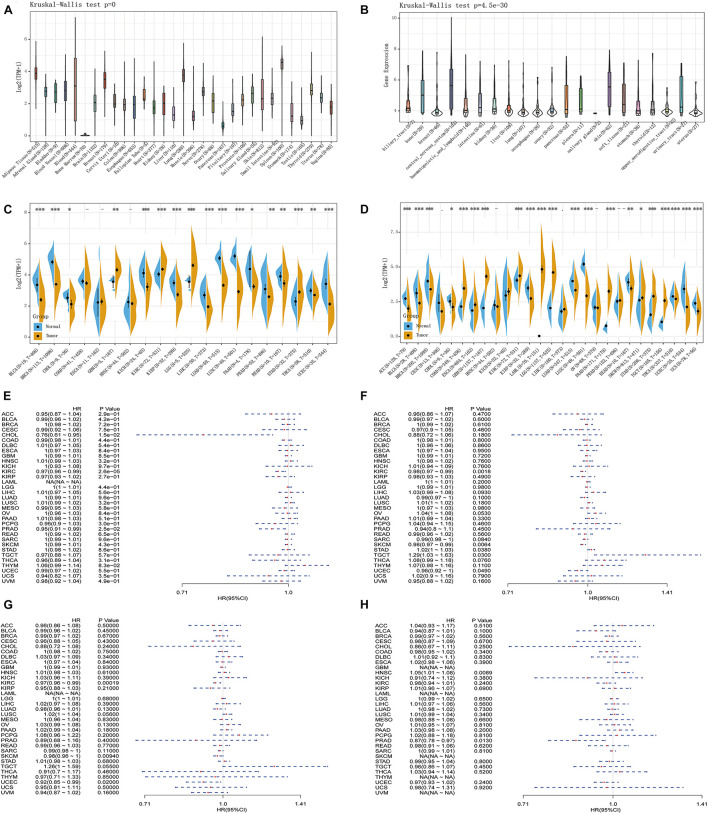
Comprehensive analysis of the expression of Toll-like receptor 4 (*TLR4*) gene in various databases. The Genotype-Tissue Expression (GTEx) data set showed that the *TLR4* gene was basically not expressed in bone marrow or 31 other tissues and was most highly expressed in the spleen **(A)**. The CCLE database (*n* = 1,019) showed that the *TLR4* gene expression was lowest in salivary glands and highest in the central nervous system **(B)**. There are few normal tissue data in The Cancer Genome Atlas (TCGA) database **(C)**. The GTEx database (*n* = 6,678) and the tumor data in TCGA database (*n* = 9,498) are compared and displayed **(D)**. Compared with normal tissues, *TLR4* gene expression is low in ACC, BLCA, BRCA, CESC, CHOL, HNSC, KIRP, LUAD, READ, HCA, UCEC, and UCS. In some data sets, COAD, ESCA, GBM, KICH, LGG, LIHC, PAAD, SKCM, STAD, and TGCT have high expressions. PFI showed that *TLR4* was notably related to the prognosis of CHOL (*p* = 0.015), KIRC (*p* = 2.6e-5), and PRAD (*p* = 0.025) **(E)**. OS displayed that *TLR4* was markedly correlated with the prognosis of KIRC (*p* = 0.0018), SKCM (*p* = 0.0064), STAD (*p* = 0.038), TGCT (*p* = 0.03), and UCEC (*p* = 0.049) **(F)**. DSS reflected that *TLR4* was observably correlated with the prognosis of KIRC (*p* = 0.00019), SKCM (*p* = 0.0094), and UCEC (*p* = 0.02) **(G)**. The prognosis of DFI showed that *TLR4* was dramatically correlated with the prognosis of HNSC (*p* = 0.0089) and PRAD (*p* = 0.013) **(H)**. A *p*-value <0.05 was considered statistically significant. The symbols “^∗^,” “^∗∗^,” and “^∗∗∗^” refer to *p*-values <0.05, <0.01, and <0.001, respectively.

The analysis of the GTEx data set showed that the *TLR4* gene was basically not expressed in bone marrow or 31 other tissues and was most highly expressed in the spleen (Figure 1A). The CCLE database (*n* = 1,019) analysis showed that among 21 tumor cell lines, *TLR4* gene expression was lowest in salivary glands and highest in the central nervous system (Figure 1B). Furthermore, because there are few normal tissue data in TCGA database (Figure 1C), we then compared the normal tissue data in the GTEx database (*n* = 6,678) to the tumor data in TCGA database (*n* = 9,498) (Figure 1D). Compared with normal tissues, *TLR4* gene is low expression in adrenocortical carcinoma (ACC), bladder urothelial carcinoma (BLCA), BRCA, cervical squamous cell carcinoma and endocervical adenocarcinoma (CESC), cholangiocarcinoma (CHOL), head and neck squamous cell carcinoma (HNSC), kidney renal papillary cell carcinoma (KIRP), lung adenocarcinoma (LUAD), rectal adenocarcinoma (READ), thyroid cancer (THCA), UCEC, and uterine carcinoma (UCS). In some data sets, COAD, esophageal carcinoma (ESCA), glioblastoma multiforme (GBM), kidney chromophobe (KICH), brain lower grade glioma (LGG), LIHC, PAAD, SKCM, STAD, and TGCT have high *TLR4* gene expression.

### The Prognosis and Toll-Like Receptor 4 Gene Expression in 33 Tumors

**FIGURE 2:**
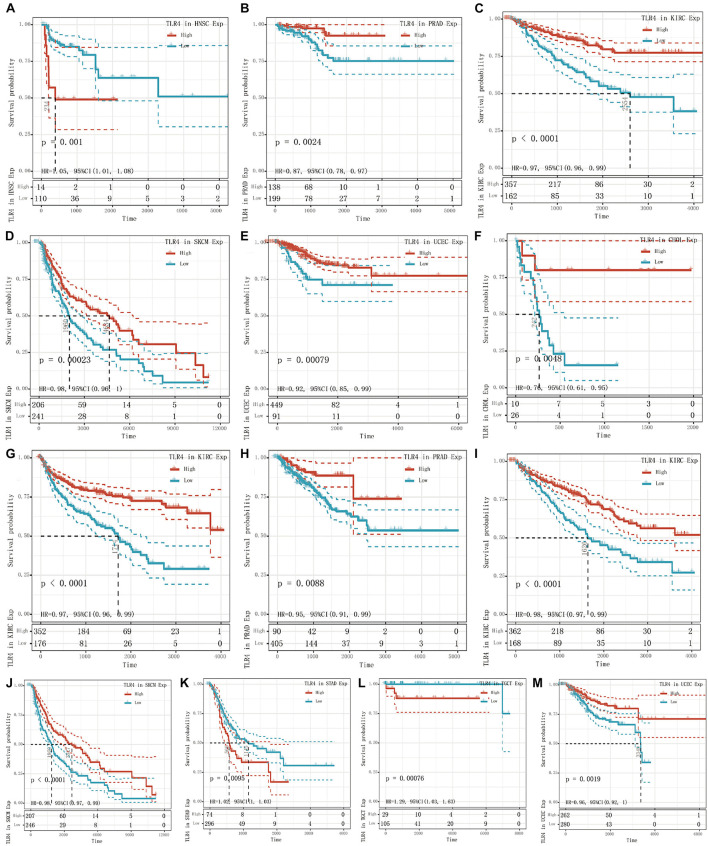
Survival curve of cancer types with significant correlation between *TLR4* gene expression and prognosis. The low expression of *TLR4* was related to the low survival rate of KIRC (*p* < 0.0001) **(C,G,I)**, SKCM (*p* = 0.00023) **(D,J)**, UCEC (*p* = 0.00079) **(E,M)**, and CHOL (*p* = 0.0048) **(F)**. High *TLR4* expression is related to low survival rates of HNSC (*p* = 0.001) **(A)**, PRAD (*p* = 0.0024) **(B,H)**, STAD (*p* = 0.0095) **(K)**, and TGCT (*p* = 0.00076) **(L)**.

To determine the correlation of *TLR4* gene expression with the patient prognosis in 33 tumors, we used gene expression profile data and single-factor regression analysis to draw forest plots (*p* < 0.05). PFI showed that *TLR4* was notably related to the prognosis of CHOL (*p* = 0.015), KIRC (*p* = 2.6e-5), and PRAD (*p* = 0.025) (Figure 1E). OS displayed that *TLR4* was markedly correlated with the prognosis of KIRC (*p* = 0.0018), SKCM (*p* = 0.0064), STAD (*p* = 0.038), TGCT (*p* = 0.03), and UCEC (*p* = 0.049) (Figure 1F). DSS reflected that *TLR4* was correlated with the prognosis of KIRC (*p* = 0.00019), SKCM (*p* = 0.0094), and UCEC (*p* = 0.02) (Figure 1G). DFI showed that *TLR4* was dramatically correlated with the prognosis of HNSC (*p* = 0.0089) and PRAD (*p* = 0.013) (Figure 1H). These outcomes showed that low expression of *TLR4* is related to the low survival rate of KIRC (*p* < 0.0001) (Figures 2C,G,I), SKCM (*p* = 0.00023) (Figures 2D,J), UCEC (*p* = 0.00079) (Figures 2E,M), and CHOL (*p* = 0.0048) (Figure 2F), while high *TLR4* expression is related to low survival rates of HNSC (*p* = 0.001) (Figure 2A), PRAD (*p* = 0.0024) (Figures 2B,H), STAD (*p* = 0.0095) (Figure 2K), and TGCT (*p* = 0.00076) (Figure 2L). Finally, *TLR4* gene expression and OS-related cancer types were screened for further study. Therefore, the low *TLR4* expression may be a risk factor for an inferior prognosis in patients with KIRC, SKCM, UCEC, and CHOL, and similarly, high *TLR4* expression may be a risk factor for an inferior prognosis in patients with HNSC, PRAD, STAD, and TGCT.

### Relationship Between Toll-Like Receptor 4 Expression and Six Types of Infiltrating Immune Cells

**FIGURE 3:**
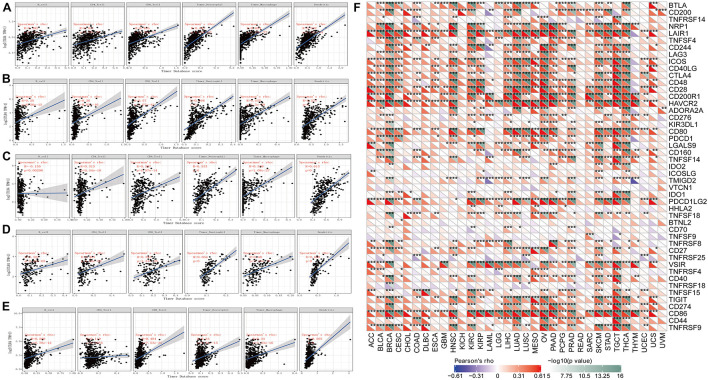
Correlation analysis of *TLR4* gene expression in immune infiltration, immune checkpoint, and in 33 tumors. **(A–E)** The expression of *TLR4* was negatively correlated with the expression of B cells in STAD, KIRC, UCEC, TGCT, and SKCM. While the expression of *TLR4* was positively correlated with the infiltration of B cells, CD4+ T cells, CD8+ T cells, neutrophils, macrophages, and dendritic cells in STAD, KIRC, UCEC, TGCT, and SKCM. **(F)** The results showed that the five prognostic-related cancers of KIRC, SKCM, STAD, TGCT, and UCEC are highly positively correlated with the six immune checkpoints of ICOS, CTLA4, CD28, CD80, PDCD1LG2, and CD86 (*r* > 0, ***p < 0.001).

Tumor-infiltrating lymphocytes are self-governed predictors of tumor sentinel lymph node status and survival rate (Dunn et al., 2007; Azimi et al., 2012). We analyzed the expression of *TLR4* and six types of infiltrating immune cells (B cells, CD4+ T cells, CD8+ T cells, neutral granulocytes, macrophages, and dendritic cells). The outcomes indicated that the expression of *TLR4* was negatively correlated with the expression of B cells in STAD, KIRC, UCEC, TGCT, and SKCM. The expression of *TLR4* was positively correlated with the infiltration of B cells, CD4+ T cells, CD8+ T cells, neutrophils, macrophages, and dendritic cells in STAD, KIRC, UCEC, TGCT, and SKCM (Figures 3A–E). To make the results more convincing, we conducted more in-depth research on tumor samples. The results revealed that the expression of *TLR4* gene in KIRC, TGCT, UCEC, SKCM and STAD has different degrees of correlation with tumor infiltrating immune cell subsets (Supplementary Figures 1–3). These findings strongly indicated that the *TLR4* gene plays a role in the immune infiltration of KIRC, SKCM, STAD, TGCT, and UCEC.

**FIGURE 4:**
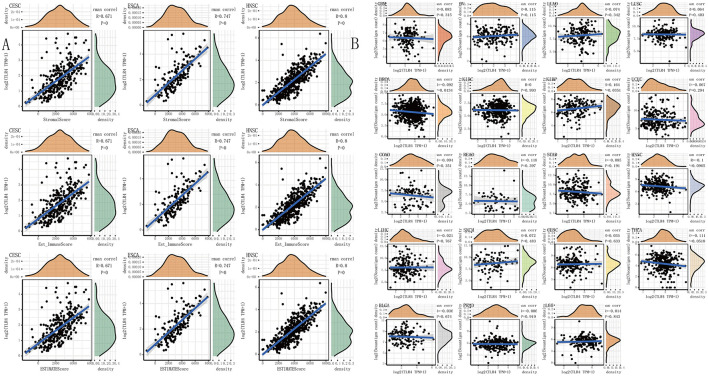
The relationship between the *TLR4* gene expression and immune score, neoantigens. **(A)** The expression of *TLR4* gene in the ESTIMATE immune score was concerned with CESC (*p* = 0), ESCA (*p* = 0), HNSC (*p* = 0) are prominently correlated. **(B)** KIRC, SKCM, STAD, TGCT, and UCEC were not notably related to neoantigens. A *p*-value <0.05 was considered statistically significant. The symbols “*,” “**,” and “***” refer to *p*-values <0.05, <0.01, and <0.001, respectively.

An increasing number of reports have proven that the tumor immune microenvironment plays a part in the occurrence and development of tumors. To further evaluate the role of *TLR4* in the tumor immune microenvironment, our results showed that the expression of the *TLR4* gene in the ESTIMATE immune score was associated with CESC (*p* = 0), ESCA (*p* = 0), and HNSC (*p* = 0) (Figure 4A). These results showed that the five prognosis-related cancers—KIRC, SKCM, STAD, TGCT, and UCEC—were highly positively correlated with the six immune checkpoints—inducible T-cell costimulator (ICOS), cytotoxic T lymphocyte-associated molecule 4 (CTLA4), CD28, CD80, PDCD1LG2, and CD86 (*r* > 0, ^∗∗∗^*p* < 0.001) (Figure 3F and Table 1).

**TABLE 1:** The relationship between *TLR4* gene expression in KIRC, SKCM, STAD, TGCT, UCEC and immune checkpoint.

R	Cancer				
	
	KIRC	SKCM	STAD	TGCT	UCEC
ICOS	0.39	0.47	0.35	0.70	0.24
CTLA4	0.22	0.36	0.21	0.60	0.23
CD28	0.44	0.44	0.43	0.67	0.25
CD80	0.44	0.52	0.40	0.68	0.10
PDCD1LG2	0.56	0.39	0.51	0.67	0.22
CD86	0.60	0.65	0.58	0.76	0.22

*The *P* value of immune checkpoints (ICOS, CTLA4, CD28, CD80, PDCD1LG2, and CD86) is less than 0.001.*

### The Relationship Between Toll-Like Receptor 4 Gene Expression in Immunotherapy-Related Markers and Clinicopathological Characteristics

**FIGURE 5:**
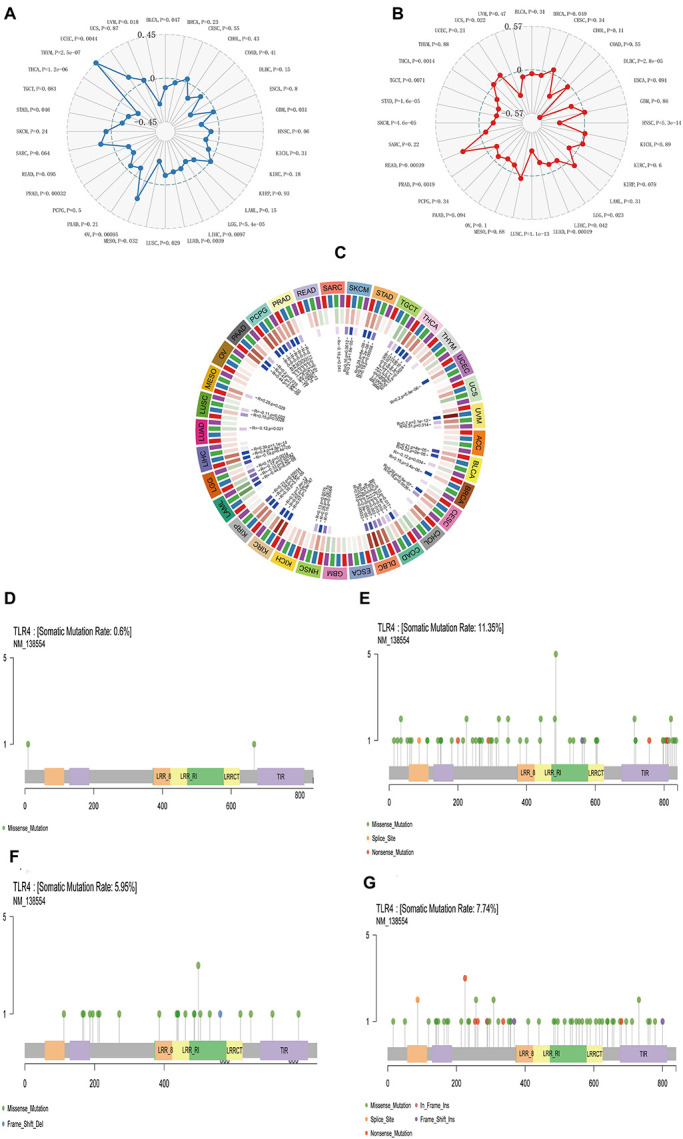
The relationship between *TLR4* gene expression and TMB, MSI, methyltransferase, and mutation frequency. **(A)** The expression of *TLR4* gene was noticeably relevant to TMB in STAD (*p* = 0.046) and UCEC (*p* = 0.0044). **(B)** The expression of *TLR4* gene in TGCT (*p* = 0.0071), STAD (*p* = 1.6e-05), and SKCM (*p* = 4.6e-05) was significantly correlated with MSI. **(C)** The analysis outcomes revealed that the expression of *TLR4* gene was conspicuously linked to methyltransferase DNMT2 and DNMT3B in SKCM and STAD (*p* < 0.05) and was markedly correlated with methyltransferase DNMT2 in UCEC and KIRC (*p* < 0.05). TCGA analysis displayed that the mutation rates of *TLR4* gene in KIRC **(D)**, SKCM **(E)**, STAD **(F)**, and UCEC **(G)** were 0.6, 11.35, 5.95, and 7.74%, respectively.

We separately counted the number of neoantigens in each tumor sample, and the results showed that KIRC, SKCM, STAD, TGCT, and UCEC were not notably related to neoantigens (Figure 4B). TMB is a quantitative biomarker that can reflect the number of mutations in tumor cells. We separately counted the TMB of KIRC, SKCM, STAD, TGCT, and UCEC samples, and the results showed that the expression of the *TLR4* gene was noticeably related to TMB in STAD (*p* = 0.046) and UCEC (*p* = 0.0044) (Figure 5A). Our research results displayed that the expression of the *TLR4* gene in TGCTs (*p* = 0.0071), STAD (*p* = 1.6e-05), and SKCM (*p* = 4.6e-05) was significantly correlated with MSI (Figure 5B). The analysis outcomes revealed that the expression of the *TLR4* gene was conspicuously linked to the methyltransferases DNA methyltransferase 2 (DNMT2) and DNA methyltransferase 3B (DNMT3B) in SKCM and STAD (*p* < 0.05) and was markedly correlated with the methyltransferase DNMT2 in UCEC and KIRC (*p* < 0.05) (Figure 5C). The above results suggest that *TLR4* gene expression has a certain effect on the prognosis of patients with KIRC, SKCM, STAD, TGCT, and UCEC. Moreover, we also analyzed the mutation patterns of the *TLR4* gene in tumor samples. TCGA analysis revealed that the mutation rates of the *TLR4* gene in KIRC (Figure 5D), SKCM (Figure 5E), STAD (Figure 5F), and UCEC (Figure 5G) were 0.6, 11.35, 5.95, and 7.74%, respectively. In addition, we further explored the somatic mutations of *TLR4* gene in KIRC, SKCM, STAD, TGCT, and UCEC and the relationship between *TLR4* gene expression and age, gender, race and stage. The results showed that the *TLR4* gene somatic mutations in KIRC, SKCM, STAD, TGCT and UCEC are mainly diploid/normal, arm-level deletion, arm-level gain, and high amplification rarely (Supplementary Figure 4). Moreover, the expression of *TLR4* gene is correlated with gender and race of TGCT, KIRC, SKCM and UCEC, and correlated with the age of KIRC. In addition, the expression of *TLR4* gene is correlated with the tumor stages of KIRC and UCEC (Supplementary Figure 5). Except for TGCT and UCEC, the expression of *TLR4* gene in KIRC, SKCM and STAD tumor stages is consistent with the results of previous studies (Supplementary Figure 6).

**FIGURE 6:**
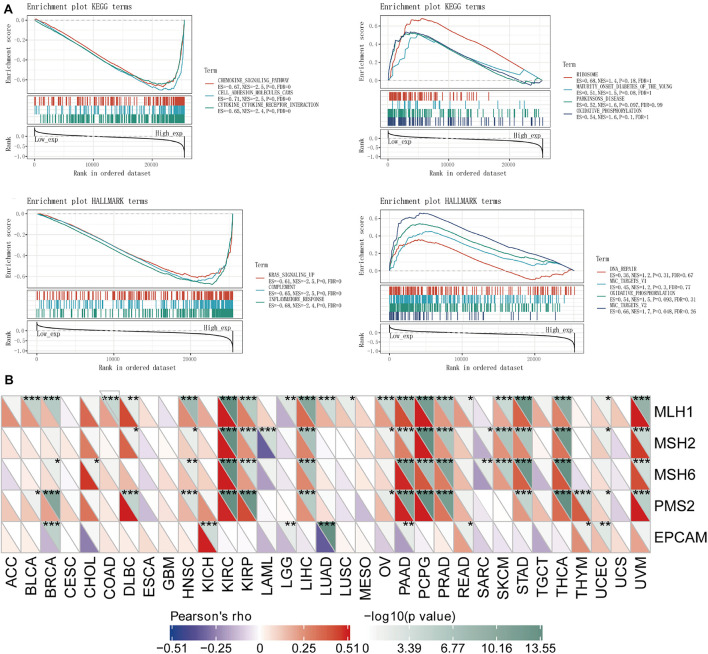
Analysis of the *TLR4* gene enrichment and DNA repair genes. **(A)** KEGG analysis displayed that *TLR4* was highly expressed in chemokine signaling pathway (*p* = 0), cell adhesion molecule (*p* = 0), and interaction of cytokine receptor (*p* = 0). HALLMARK analysis showed that *TLR4* is lowly expressed in MYC_TARGETS_V2 (*p* = 0.048) and highly expressed in KRAS signaling pathway (*p* = 0) and inflammatory response (*p* = 0) pathway. **(B)** The *TLR4* gene expression was highly linked to DNA repair genes MLH1, MSH2, MSH6, and PMS2 in KIRC and STAD and MLH1, MSH2, and MSH6 in SKCM (**p < 0.001); mildly correlated with MLH1, MSH2, and PMS2 in UCEC (*p < 0.001) 05); and showed a moderate correlation with EPCAM (**p < 0.01). A *p*-value <0.05 was considered statistically significant.

For the purpose of better understanding the correlation and latent mechanism of *TLR4* expression in cancer, TCGA expression profile data were used to assess the relationship between five DNA repair genes (MLH1, MSH2, MSH6, PMS2, EPCAM mutations) and gene expression. Our results showed that *TLR4* gene expression was highly linked to the DNA repair genes MLH1, MSH2, MSH6, and PMS2 in KIRC and STAD and MLH1, MSH2, and MSH6 in SKCM (^∗∗^*p* < 0.001); mildly correlated with MLH1, MSH2, and PMS2 in UCEC (^∗^*p* < 0.001) 05); and moderately correlated with EPCAM (^∗∗^*p* < 0.01) (Figure 6B).

To observe the effect of gene expression on tumors, we used GSEA to analyze the enrichment of the KEGG and HALLMARK pathways in the high-expression group and the low-expression group. KEGG analysis showed that *TLR4* was highly expressed in the chemokine signaling pathway (*p* = 0), cell adhesion molecule (*p* = 0), and cytokine receptor interaction (*p* = 0). HALLMARK analysis showed that *TLR4* was expressed at low levels MYC_TARGETS_V2 cells (*p* = 0.048) and highly expressed in the KRAS signaling pathway (*p* = 0) and inflammatory response (*p* = 0) pathway (Figure 6A and Table 2).

**TABLE 2:** The expression of *TLR4* gene in tumor was analyzed by GSEA.

Term signaling pathway	ES	NES	NP	FDR	FWER
KEGG chemokine signaling pathway	–0.6741	–2.5244	0	0	0
KEGG cell adhesion molecules CAMs	–0.7094	–2.4558	0	0	0
KEGG cytokine cytokine receptor interaction	–0.6474	–2.4433	0	0	0
KEGG toll like receptor signaling pathway	–0.6781	–2.4385	0	0	0
KEGG leukocyte transendothelial migration	–0.6589	–2.4091	0	0	0
KEGG Leishmania infection	–0.8037	–2.3823	0	0	0
KEGG natural killer cell mediated cytotoxicity	–0.6407	–2.3531	0	0	0
KEGG FC gamma R mediated phagocytosis	–0.6433	–2.338	0	0	0
KEGG JAK STAT signaling pathway	–0.6001	–2.3157	0	0	0
KEGG viral myocarditis	–0.7156	–2.2852	0	0	0
KEGG FC epsilon RI signaling pathway	–0.6194	–2.2632	0	0	0
KEGG hematopoietic cell lineage	–0.7097	–2.236	0	0	0
KEGG B cell receptor signaling pathway	–0.665	–2.2248	0	0	0
KEGG T cell receptor signaling pathway	–0.6492	–2.2132	0	0	0
KEGG vascular smooth muscle contraction	–0.5684	–2.2045	0	0	0
KEGG regulation of actin cytoskeleton	–0.5457	–2.1893	0	1.00E-04	0.001
KEGG autoimmune thyroid disease	–0.7856	–2.1818	0	2.00E-04	0.002
KEGG NOD like receptor signaling pathway	–0.6492	–2.178	0	2.00E-04	0.002
KEGG lysosome	–0.6472	–2.1712	0	3.00E-04	0.004
KEGG focal adhesion	–0.5785	–2.1248	0	8.00E-04	0.011
KEGG apoptosis	–0.5731	–2.1239	0	8.00E-04	0.011
KEGG MAPK signaling pathway	–0.4922	–2.1019	0	9.00E-04	0.015

*ES, Enrichment score; NES, standardized enrichment score; NP, nominal p-value; FDR, false discovery rate; FWER, Family-wise error rate.*

**FIGURE 7:**
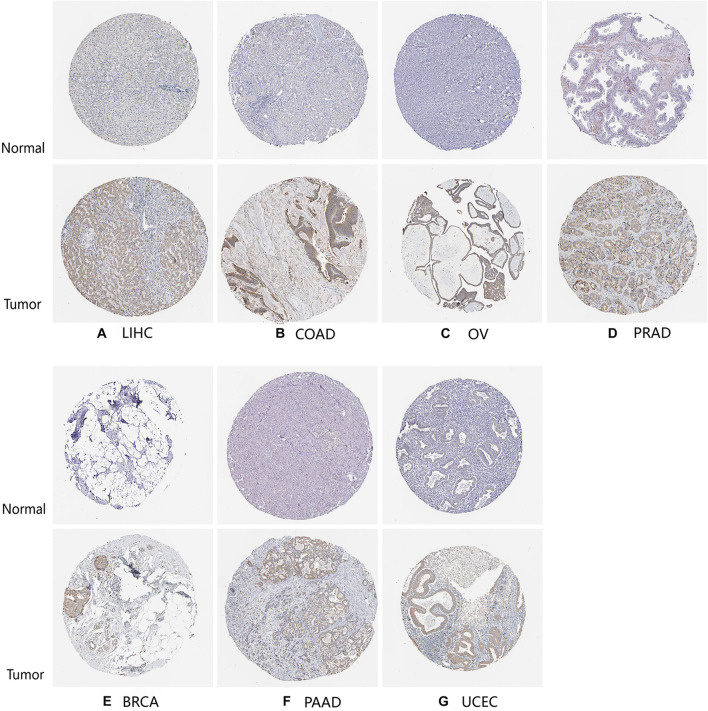
HPA database verifies the expression of *TLR4* gene in seven tumors. The expression of *TLR4* gene in LIHC **(A)**, COAD **(B)**, OV **(C)**, PRAD **(D)**, BRCA **(E)**, PAAD **(F)**, and UCEC **(G)** is significantly higher than that in the corresponding normal tissues.

**FIGURE 8:**
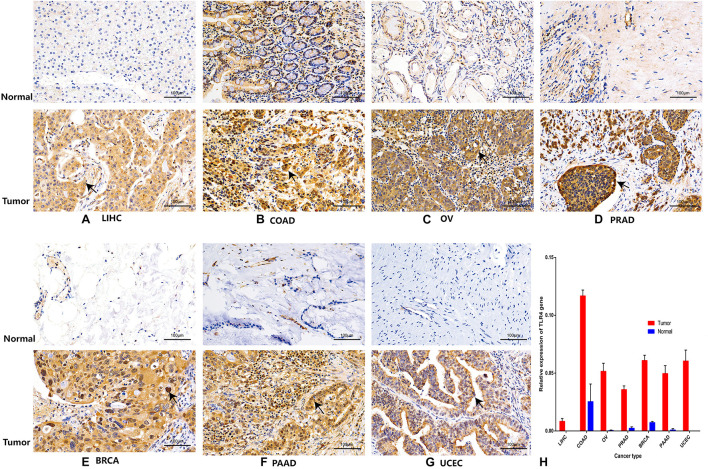
Immunohistochemistry verifies the expression of *TLR4* gene in seven tumors. The expression of *TLR4* gene in LIHC **(A)**, COAD **(B)**, OV **(C)**, PRAD **(D)**, BRCA **(E)**, PAAD **(F)**, and UCEC **(G)** is significantly higher than that in the corresponding normal tissues.

Finally, to further compare whether the expression of the *TLR4* gene is different in the cancer tissues of 14 types of tumors and the corresponding normal tissues, both the HPA database and IHC staining were verified, and the results confirmed that the *TLR4* gene was significantly highly expressed in COAD, LIHC, OV, PRAD, BRCA, PAAD, and UCEC (Figures 7, 8 and Table 3).

**TABLE 3:** Average optical density of seven kinds of cancer.

Cancer	LIHC	COAD	OV	PRAD	BRCA	PAAD	UCEC
Mean density	Mean ± SD	Mean ± SD	Mean ± SD	Mean ± SD	Mean ± SD	Mean ± SD	Mean ± SD
Tumor	0.0083 ± 0.00252**	0.1168 ± 0.00503**	0.0516 ± 0.00669**	0.0358 ± 0.00316**	0.061 ± 0.0045**	0.0497 ± 0.00679**	0.0605 ± 0.00945**
Normal	0 ± 0.00006	0.0254 ± 0.01518	0.0004 ± 0.00046	0.0024 ± 0.00119	0.0073 ± 0.00093	0.0009 ± 0.0011	0 ± 0

A *p*-value < 0.05 was considered statistically significant. The symbols “**” refer to *p*-values < 0.01. It means that there is a significant difference between cancer and normal tissue.

## Discussion

Malignant tumors are a complex disease that is related to the expression and functional changes of a variety of immune system-related molecules (Gu et al., 2016). On the one hand, the immune system plays a strong antitumor role in; on the other hand, it also contributes to the occurrence and development of tumors, so it is thought to be a double-edged sword (Hagemann et al., 2007). Previous studies have indicated that tumor progression, drug resistance, and metastasis are caused by cytokines and chemokines secreted by tumor cells and their adjacent stromal cells. In previous experiments, it has been shown that cancer cells are usually highly infiltrated by innate immune cells, and they have co-opted some immune-related pathways during the development of the disease (He et al., 2007). Recently, the important role of the *TLR4* pathway in drug resistance and metastasis has drawn the attention of scientists (González-Reyes et al., 2010).

*TLR4* belongs to the pathogen recognition receptor (PRR) family, and it plays an important role in activating/inhibiting immune and non-immune cells by recognizing pathogen-related molecular patterns (PAMPs) and damage-related molecular patterns (DAMPs; Bagheri et al., 2014). Extracellular *TLR4* contains a leucine repeat sequence (LRR) domain that binds to a ligand, and it can recognize a variety of ligands such as lipopolysaccharide (LPS) and heat shock proteins 60 and 70 (Evans et al., 2003). It interacts with MYD88 and TRIF through the cytoplasmic Toll/IL-1 receptor (TIR) domain (Yamamoto et al., 2003). In the MYD88-dependent pathway, *TLR4* and myeloid differentiation 2 produce dimers and then recruit the adaptor protein [toll-interleukin 1 receptor domain-containing adaptor protein (TIRAP)] containing the TIRAP domain and the two adaptor proteins of MYD88. The activation of MYD88 in turn leads to the phosphorylation of interleukin-1 receptor-associated kinase-4 (IRAK-4) and IRAK-1, and tumor necrosis factor (TNF) receptor-associated factor-6 (TRAF-6) is the main transcription factor molecule that activates the nuclear factor-κB (NF-κB) chain enhancer of B cells. After activation, both of these proteins stimulate the κB-inhibitor (IκB) complex (IKK) and degrade IκBα. This leads to NF-κB activation and nuclear translocation, thereby altering the cell function (Yamamoto et al., 2003).

In the process of tumor initiation or progression, different tumors may have different degrees of the *TLR4* involvement. Based on these facts, we decided to study the expression of *TLR4* gene across cancers and its relationship with the prognosis of different cancer patients and tumor-infiltrating lymphocytes. To the best of our knowledge, this is the first comprehensive and systematic study of the differential expression and related mechanisms of *TLR4* across cancers.

Here, we analyzed the expression and prognosis of the *TLR4* gene in 33 tumors in TCGA database using gene expression profile data. Differential expression of *TLR4* between cancer cells and normal tissues existed in many types of cancer. We found that compared with normal tissues, *TLR4* gene expression was lower in ACC, BLCA, BRCA, CESC, CHOL, HNSC, KIRP, LUAD, READ, THCA, UCEC, and UCS. In some data sets, high expression was observed in COAD, ESCA, GBM, KICH, LGG, LIHC, PAAD, SKCM, STAD, and TGCT. In previous experiments, it was proven that the genes contained in this feature are all related to cancer (Schmausser et al., 2004; Hassan et al., 2006; He et al., 2007; Ikebe et al., 2009; Szczepanski et al., 2009; Tang and Zhu, 2012; Tewari et al., 2012; Wang et al., 2014; Jain et al., 2015; Olbert et al., 2015; Lin et al., 2016; Jiang et al., 2017). However, in these databases, we also found that low *TLR4* expression was associated with a poor prognosis in KIRC, SKCM, and UCEC. Interestingly, high *TLR4* expression was associated with an inferior prognosis of HNSC, PRAD, STAD, and TGCT. The expression level of the *TLR4* gene was also related to the OS of KIRC, SKCM, STAD, TGCT, and UCEC, which indicated that *TLR4* expression could be used as a predictor of tumor prognosis.

Another vital aspect of this study is that the *TLR4* gene is associated with different levels of immune infiltration in cancers (KIRC, SKCM, STAD, TGCT, and UCEC), and this is further associated with OS. Through time analysis of public databases, our results revealed that *TLR4* gene expression was markedly negatively correlated with B cells in STAD and positively mutually related to the infiltration levels of CD4+ T cells, CD8+ T cells, neutrophils, macrophages, and dendritic cells. The expression of the *TLR4* gene was related to B cells, CD4+ T cells, CD8+ T cells, and neutrophils in KIRC, UCEC, TGCT, and SKCM. The infiltration levels of the cells, macrophages and dendritic cells, were significantly positively correlated. These results suggested that *TLR4* gene can regulate tumor immunity in KIRC, SKCM, STAD, TGCT, and UCEC. In this era of stratified medicine, the identification of immune biomarkers is increasingly vital (Chen et al., 2019). A number of studies have found that tumor-infiltrating lymphocytes, namely, tumor-associated macrophages (TAMs) and tumor-infiltrating neutrophils (TINs), influence the prognosis and the efficacy of chemotherapy and immunotherapy (Waniczek et al., 2017; Zhang et al., 2018). Therefore, our findings may play a crucial role in identifying new immune-related therapeutic targets.

Furthermore, immune checkpoints are molecules that have the ability to regulate T cells. They are a set of inhibition and stimulation pathways that directly affect the function of immune cells (Feng et al., 2019). When cancer occurs, the immune checkpoint (IC) signal becomes a way for the tumor to engage in immune escape, leading to tumor aggressiveness. To date, the most effective immune checkpoint inhibitor is a monoclonal antibody that binds to programmed death-1 (*PD-1*) or its ligand *PD-L1* (Traini et al., 2019). However, only a small number of patients receiving immune-checkpoint inhibitors (ICIs) targeting *PD-L1* or *CTLA-4* have good clinical treatment effects (Sun et al., 2018). Therefore, it is necessary to study new suppression checkpoints and their target molecules to expand the use and efficacy of existing immune checkpoint suppression therapies (Granier et al., 2017). In this study, we conducted a systematic analysis of more than 40 common immune checkpoint genes. The results showed that the five *TLR4* prognosis-related cancers KIRC, SKCM, STAD, TGCT, and UCEC were related to *ICOS*, *CTLA4*, *CD28*, and *CD80*. The six immune checkpoints, *PDCD1LG2*, and *CD86* are highly correlated. To our knowledge, previous studies have not reported a comprehensive checkpoint analysis that is highly relevant in KIRC, SKCM, STAD, TGCT, and UCEC. Immune checkpoints have triggered a wave of research on immune checkpoint therapy, that is, CD8 tumor-infiltrating T lymphocytes (TILs) are reactivated, which is a promising anticancer treatment. Hauzenberger et al. (1999) proved that TNF regulates the expression of CXCL12 through *TLR4*, thereby impairing the function of CD8 TILs. By regulating CD8 TILs, it helps to evade antitumor immunity. This mechanism seems to be related to human breast cancer (Hauzenberger et al., 1999). Similarly, a study conducted by Beswick et al. (2014) discussed the immune tolerance of colonic mucosa induced by *TLR4*/*PD-L1*. In addition, Wölfle et al. (2011) described a similar induction effect of *TLR4* activation on *PD-L1* expression. Given that the expression of *TLR4* is significantly related to the prognosis of KIRC, SKCM, STAD, TGCT, and UCEC, and previous studies have paved the way for the discovery of new checkpoints and follow-up studies, our research can provide a foundation for the development of more immunotherapies and model animals in the future and could provide potential therapeutic targets.

According to most reports, the efficacy of immune checkpoint blockade is related to the immunogenicity of the tumors (Snyder et al., 2014). Because the number of T cells infiltrated by tumors is small, tumors with low immunogenicity tend to respond weakly to immune checkpoint-blocking therapy (Tumeh et al., 2014). Under normal circumstances, immune checkpoint molecules downregulate activation signals from costimulatory molecules to maintain self-tolerance and prevent autoimmunity. However, tumor cells can use this mechanism to inhibit the activation and functional status of T cells, leading to T-cell exhaustion and tumor immune escape. Previous studies have shown that dual CD40–*TLR4* activation in a tumor is one way to overcome resistance to PD-1 blockade. The unique feature of this method is that it can lead to the loss of depleted T cells in the tumor (Khalil et al., 2019). Recent studies have shown that silica nanoparticles promote the tumor infiltration of CD8+ cytotoxic T lymphocytes (CTLs) by targeting the *TLR4*/NF-κB pathway. Through PD-1 blockade, the depletion of CTLs is activated and reversed, thereby generating durable antitumor immunity (Sun et al., 2021). Recent findings in chronic inflammatory diseases and cancer indicate that T-cell failure and lymphopenia may be related to changes in the expression level of *TLR4* (Wild et al., 2012). T-cell depletion is a key obstacle to effective immunotherapy (Pauken and Wherry, 2015). Due to the *TLR4* signaling pathway and its extensive interactions, *TLR4* is considered to be a potential target of immune agents. In view of the differences in immune efficacy caused by individual differences, it is very important to choose the most suitable therapy for each patient. Therefore, there is still much work to be done in the field of effective immunotherapy. Future research will need to fully clarify how TLR agonists affect the depletion of T cells, and the therapeutic effect on distant tumors is a very interesting issue.

In view of the correlation between *TLR4* expression and the immune invasion level and immune checkpoints in different types of cancer, we determined the relationship between *TLR4* and the prognosis of different types of cancer immune neoantigen, TMB, MSI, DNA repair genes, and DNA methylation. As expected, our results demonstrated that there was no obvious correlation with the new tumor antigen. The expression of *TLR4* gene was correlated with the target cancers. These findings may help to understand the special role of *TLR4* in genetic expression and gene repair.

As a functional unit of the genome, cell signaling pathways play an important biological role in major cellular processes including cancer occurrence and development. Bioinformatics enrichment analysis showed that *TLR4* was highly expressed in the interaction pathways of chemokine signaling pathways, cell adhesion molecules, and cytokine receptors. HALLMARK analysis showed that *TLR4* was expressed at low levels in MYC_TARGETS_V2 and that *TLR4* was expressed in the KRAS signaling pathway and highly expressed in the inflammatory response pathway. The *MYC* gene family consists of three members, *C-MYC*, *L-MYC*, and *N-MYC*. Among them, *C-MYC* is the most widely studied, and MYC is the most frequently amplified gene (Duffy and Crown, 2021). All *MYC* proteins mainly act as transcription regulators, regulating the expression of genes involved in a variety of cellular processes, proliferation, survival, metabolism, invasion, and metastasis (Duffy and Crown, 2021). According to previous reports, among 33 different cancers, *MYC* amplification was found in 21% of patients, especially BRCA, UCEC, and OV (McAnulty and DiFeo, 2020). High *MYC* has been reported to be associated with a poor prognosis of prostate cancer and breast cancer (Maroto et al., 2017). Our hallmark analysis showed that low expression of the *TLR4* gene was enriched in the *MYC* pathway. In addition, *TLR4* gene expression was related to the prognosis of the above five cancers. Combined with the above studies, we found that there may be an indispensable link between the expression of the *TLR4* gene and *MYC*, and they may be potential therapeutic candidates for the development of new therapeutic strategies. Although it is full of challenges, there is no doubt that in-depth studies of various tumor immune states are needed to provide valuable insights for potentially effective treatment strategies. Recent research showed that chemokines and cytokines, namely, TNF-α, IL-6, and chemokine (C-C motif) ligand 2 (CCL2), have roles in the formation of the cancer microenvironment and are responsible for the migration of inflammatory cells and cancer cells (King, 2015). It is worth noting that our findings have similar results to all known studies. The *TLR4* signaling pathway and its extensive interactions seem to be potential targets for immunotherapy.

Finally, to confirm the results of the above database analysis, we verified from the HPA database that the *TLR4* gene was significantly highly expressed in COAD, LIHC, OV, PRAD, BRCA, PAAD, and UCEC. In the past few years, genetic markers have often been used to predict the outcome of various tumors (Karamichalis et al., 2016), and to a certain extent, they are even better than TNM staging and histopathological diagnosis (Chlis et al., 2018). In recent years, the expression of TLR in tumor tissues has been reported, which may provide a vital mechanism for alleviation of improper immune enhancement and immune dysfunction and lead to antitumor immune tolerance (Tang and Zhu, 2012; Fu et al., 2013; Mai et al., 2013; Wang et al., 2013). There is evidence that *TLR4* has multiple associations with cancer. Different cell lines and tissue samples from patients with head and neck, esophageal, stomach, colon and rectum, liver, pancreatic, skin, breast, ovarian, and cervical cancer showed an increase in *TLR4* expression (Mai et al., 2013).

In addition, we must admit that our analysis has potential limitations. The association between the expression of the *TLR4* gene across cancers and prognosis is based on an analysis of public databases. Therefore, our work is retrospective rather than prospective. Moreover, follow-up animal experiment verification and further multicenter, large-sample, prospective studies are required to testify the relationship between *TLR4* and patient prognosis and to seek more effective treatment strategies.

## Data Availability Statement

The datasets presented in this study can be found in online repositories. The names of the repository/repositories and accession number(s) can be found in the article/Supplementary Material.

## Ethics Statement

The studies involving human participants were reviewed and approved by Institutional Ethics Committee of the Second Affiliated Hospital of Nanchang University. The patients/participants provided their written informed consent to participate in this study.

## Author Contributions

GX designed the research. JH, JX, XF, and YL performed the experiments and analyzed the data. JH wrote the manuscript. FH and GX revised the manuscript. All authors read and approved the final manuscript.

## Conflict of Interest

The authors declare that the research was conducted in the absence of any commercial or financial relationships that could be construed as a potential conflict of interest.

## Publisher’s Note

All claims expressed in this article are solely those of the authors and do not necessarily represent those of their affiliated organizations, or those of the publisher, the editors and the reviewers. Any product that may be evaluated in this article, or claim that may be made by its manufacturer, is not guaranteed or endorsed by the publisher.

## Footnotes

1
https://cistrome.shinyapps.io/timer/

2
https://www.proteinatlas.org/

## Abbreviations

TLRs: toll-like receptors
TLR4: toll-like receptor 4
KIRC: kidney renal clear cell carcinoma
SKCM: skin cutaneous melanoma
STAD: stomach adenocarcinoma
TGCT: testicular germ cell tumor
UCEC: uterine corpus endometrial carcinoma
GTEx: Genotype-Tissue Expression
CCLE: Cancer Cell Line Encyclopedia
TCGA: The Cancer Genome Atlas
PFI: process-free intervention
OS: overall survival
DSS: disease-specific survival
DFI: disease-free intervention
TIMER: Tumor Immune Assessment Resource
TMB: tumor mutation burden
MSI: microsatellite instability
GSEA: Gene Set Enrichment Analysis
HPA: Human Protein Atlas
ACC: adrenocortical carcinoma
BLCA: bladder urothelial carcinoma
BRCA: breast invasive carcinoma
CESC: cervical squamous cell carcinoma and adenocarcinoma
CHOL: cholangiocarcinoma
HNSC: head and neck squamous cell carcinoma
KIRP: kidney renal papillary cell carcinoma
LUAD: lung adenocarcinoma
READ: rectal adenocarcinoma
THCA: thyroid cancer
UCS: uterine carcinoma
COAD: colon adenocarcinoma
ESCA: esophageal carcinoma
GBM: glioblastoma multiforme
KICH: kidney chromophobe
LGG: brain lower grade glioma
LIHC: liver hepatocellular carcinoma
PAAD: pancreatic adenocarcinoma
PRAD: prostate adenocarcinoma
OV: ovarian serous cystadenocarcinoma
KEGG: Kyoto Encyclopedia of Genes and Genomes
TAM: tumor-associated macrophage
TIN: tumor-infiltrating neutrophil
PRR: pathogen recognition receptor
PAMPs: pathogen-related molecular patterns
DAMPs: damage-related molecular patterns
LRR: leucine-rich repeat
TIR: Toll/interleukin-1 receptor
IRAK-4: interleukin-1 receptor-associated kinase-4
TNF: tumor necrosis factor
TRAF-6: tumor necrosis factor receptor-associated factor-6
NF- κ B: nuclear factor- κ B
PD-1: programmed death-1
TIL: tumor-infiltrating T lymphocyte
CTLs: cytotoxic T lymphocytes
I κ B: κ B inhibitor.
