# Supplementary figures and images for: Differential Expression of the TLR4 Gene in Pan-Cancer and Its Related Mechanism
Source: Front Cell Dev Biol. 2021 Sep 23;9:700661. doi: 10.3389/fcell.2021.700661 (PMC8495169; doi:10.3389/fcell.2021.700661)

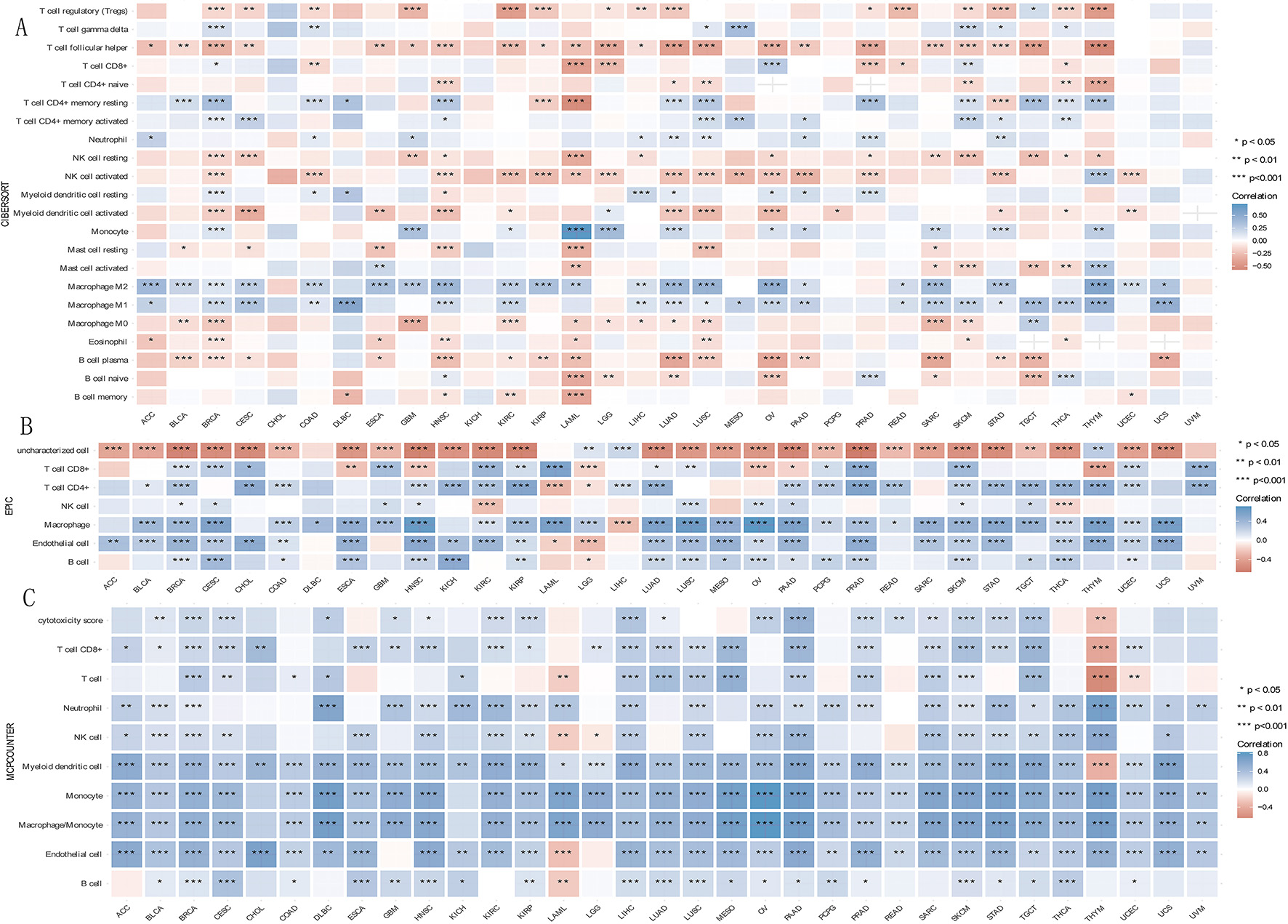

Supplement: Supplementary file 1 [file Image_1.jpg]

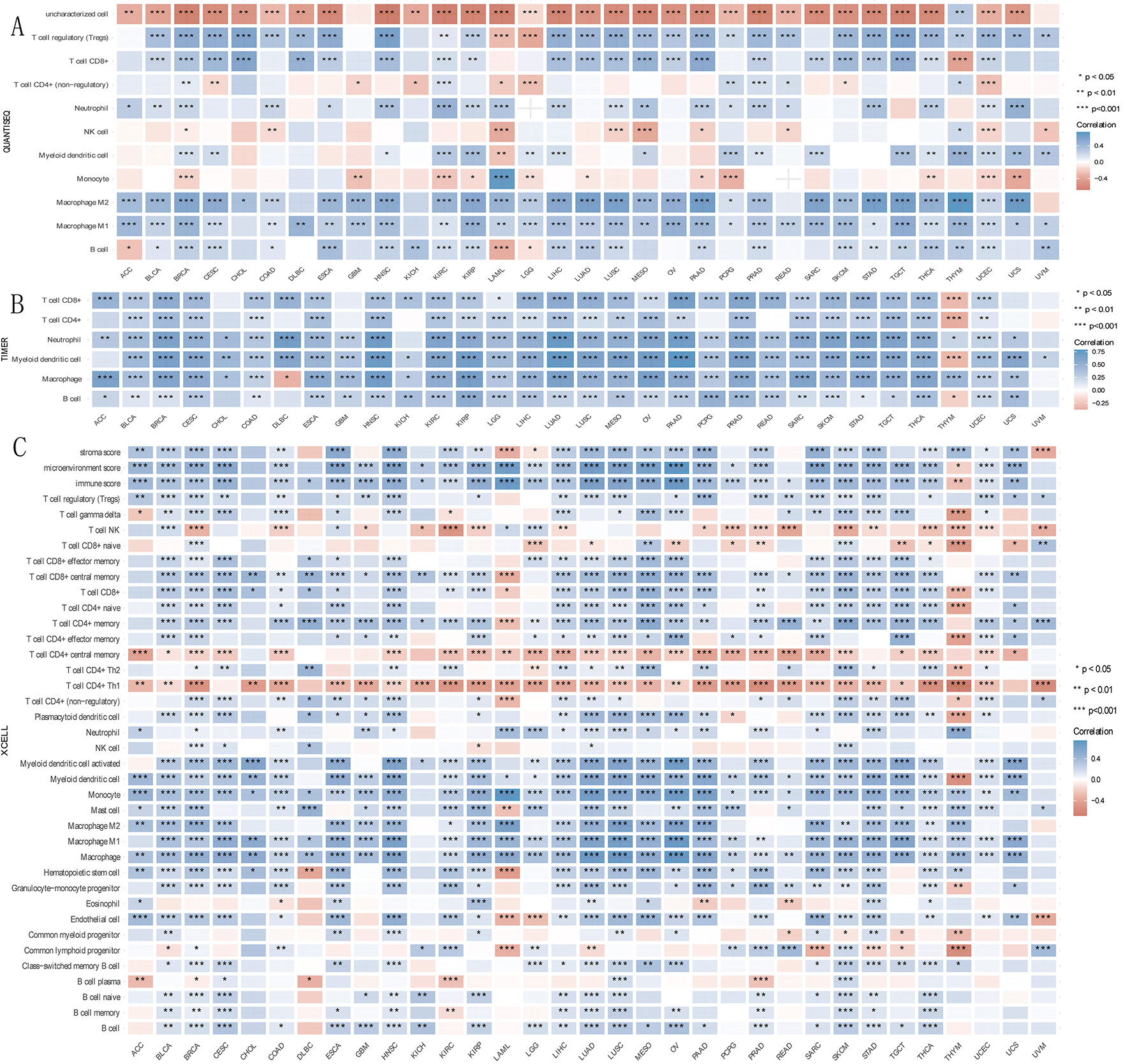

Supplement: Supplementary file 2 [file Image_2.jpg]

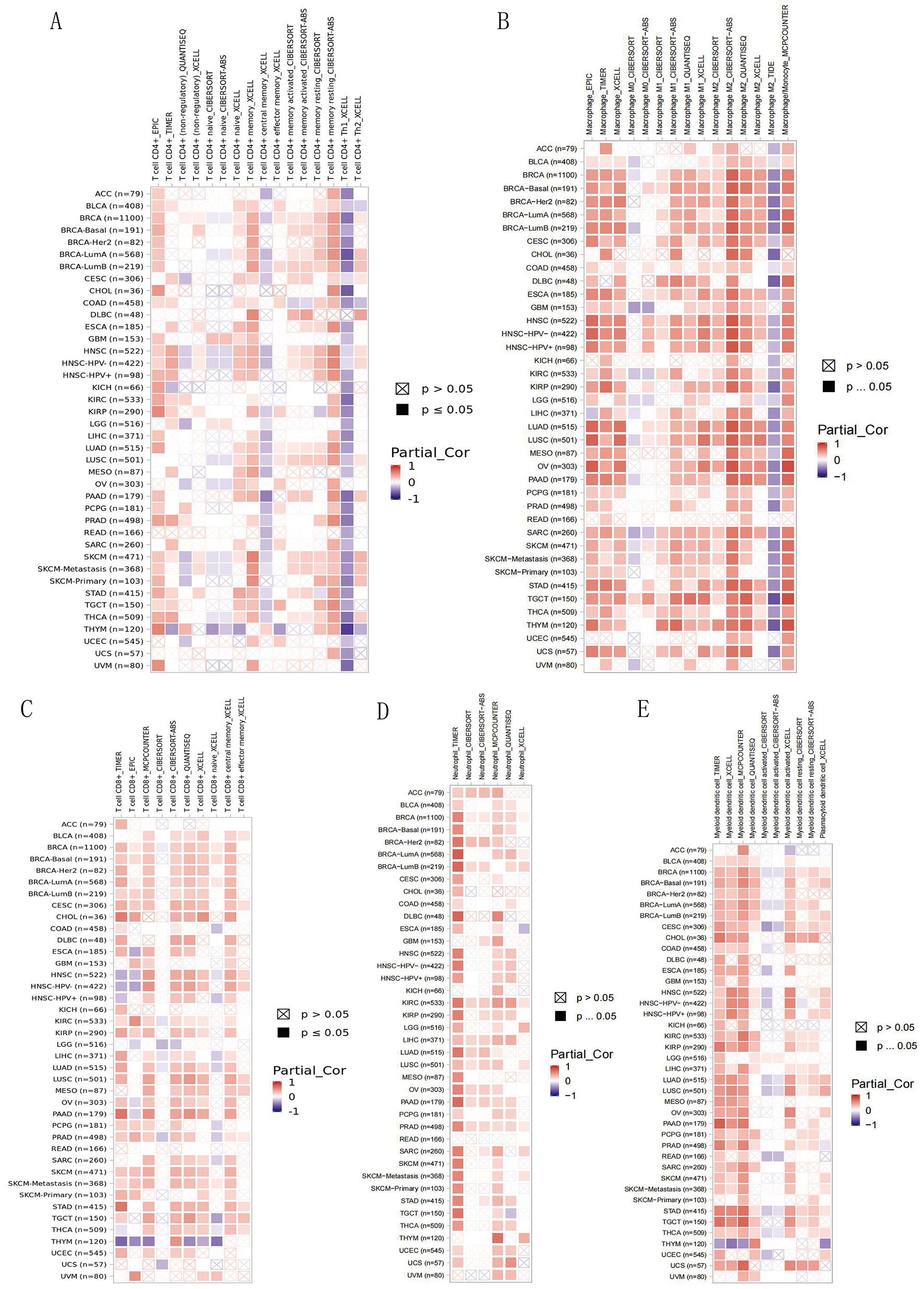

Supplement: Supplementary file 3 [file Image_3.jpg]

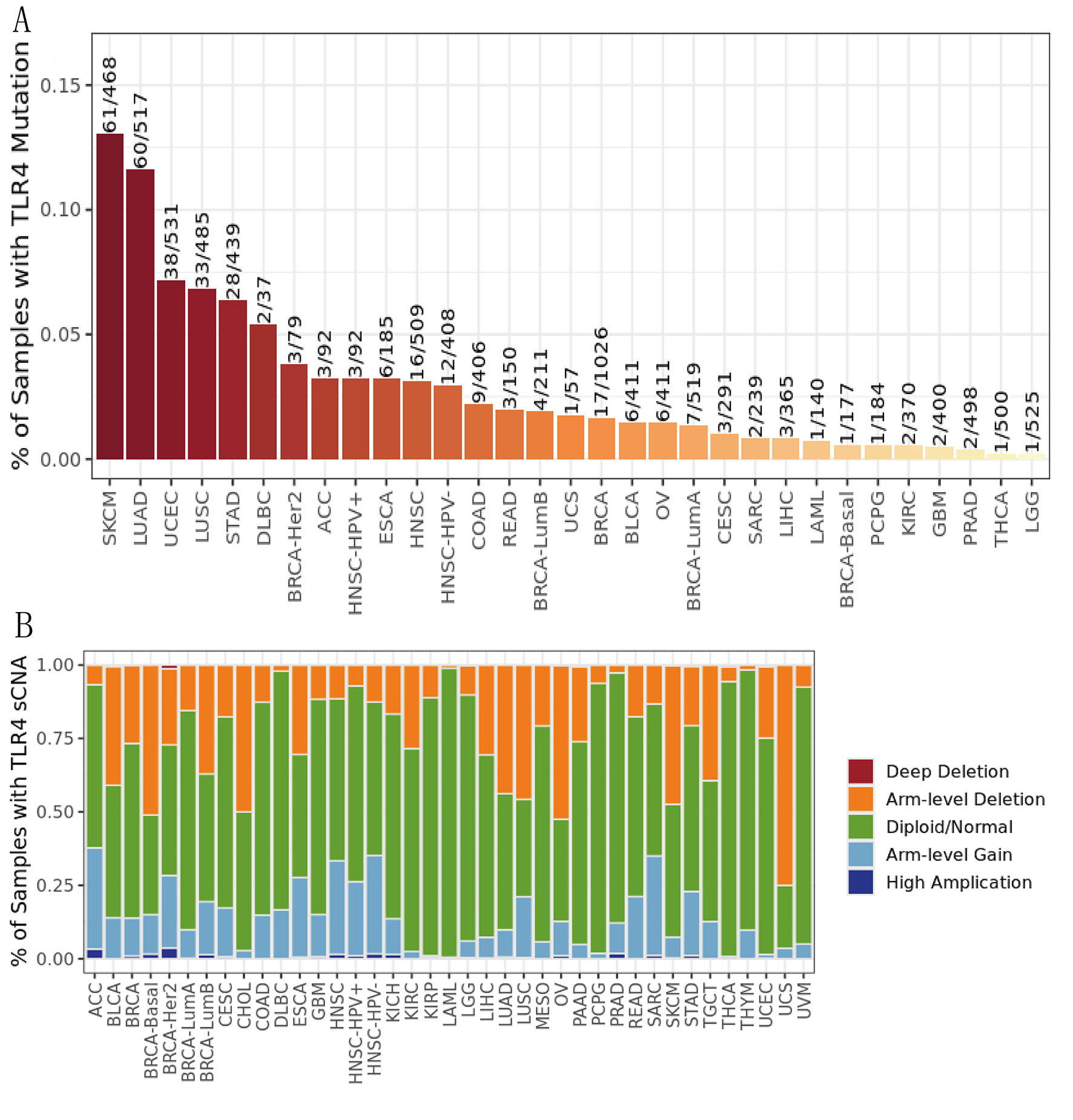

Supplement: Supplementary file 4 [file Image_4.jpg]

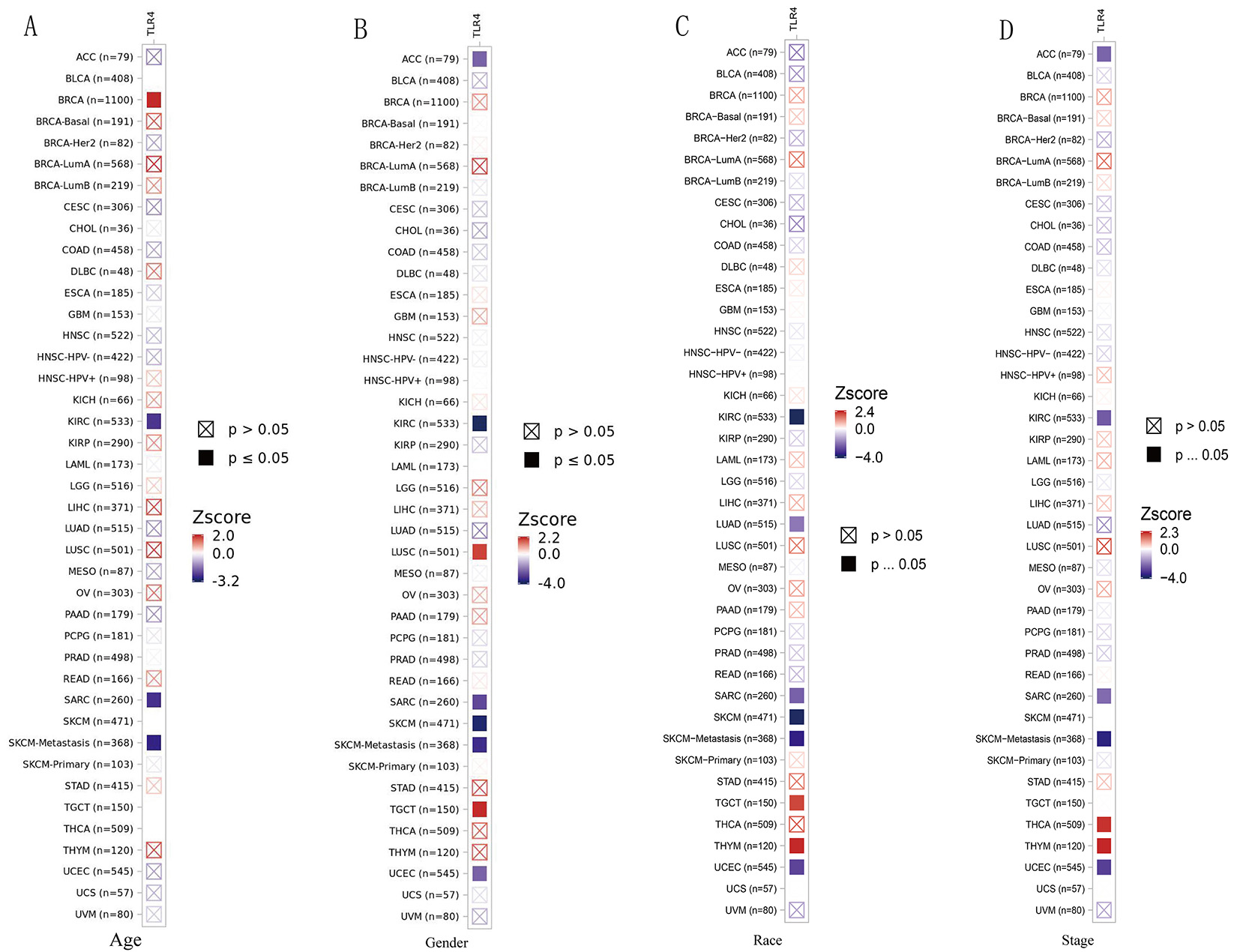

Supplement: Supplementary file 5 [file Image_5.jpg]

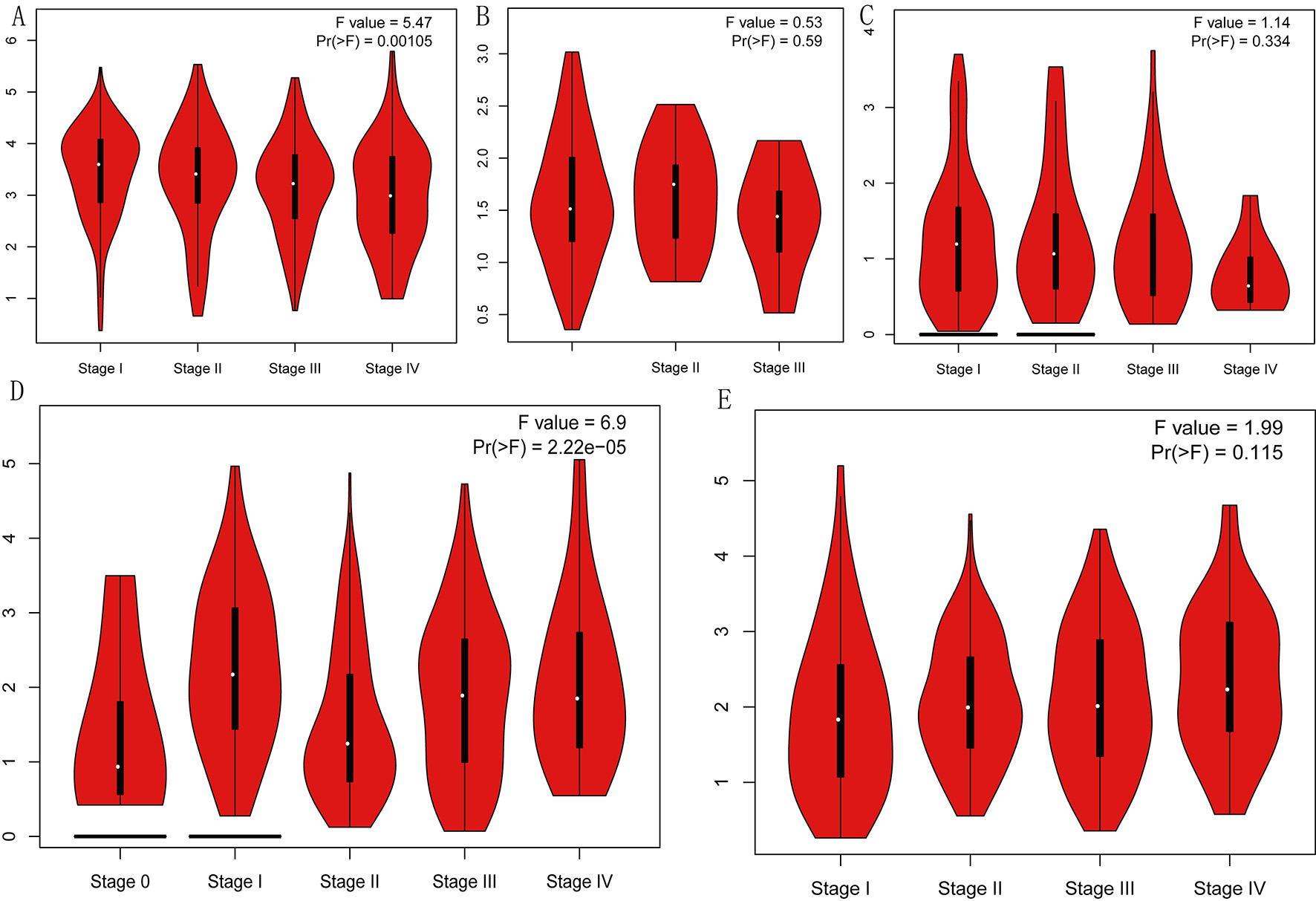

Supplement: Supplementary file 6 [file Image_6.jpg]
