# Supplementary material for: Differential Expression of the TLR4 Gene in Pan-Cancer and Its Related Mechanism
Source: Front Cell Dev Biol. 2021 Sep 23;9:700661. doi: 10.3389/fcell.2021.700661 (PMC8495169; doi:10.3389/fcell.2021.700661)
Supplement: Supplementary file 7 [file Presentation_1.pdf]

**Figure Legends**

**Figure S1** Correlation analysis of the TLR4 gene expression in KIRC, TGCT, UCEC, SKCM and STAD in MCPCOUNTER, CIBERSORT, EPIC algorithm and tumor infiltrating immune cell subsets. **(A)** Correlation analysis of the TLR4 gene expression in 5 tumors in CIBESOR algorithm and tumor infiltrating immune cell subsets. **(B)** Correlation analysis of the TLR4 gene expression in 5 tumors in EPIC algorithm and tumor infiltrating immune cell subsets. **(C)** Correlation analysis of the TLR4 gene expression in 5 tumors in MCPCOUNTER algorithm and tumor infiltrating immune cell subsets.

**Figure S2** Correlation analysis of the TLR4 gene expression in KIRC, TGCT, UCEC, SKCM and STAD in QUANTISEQ, TIMER, XCELL algorithm and tumor infiltrating immune cell subsets (see Table S1 for details). **(A)** Correlation analysis of TLR4 gene expression in 5 tumors in QUANTISEQ algorithm and tumor infiltrating immune cell subsets. **(B)** Correlation analysis of the TLR4 gene expression in 5 tumors in TIMER algorithm and tumor infiltrating immune cell subsets. **(C)** Correlation analysis of TLR4 gene expression in 5 tumors in XCELL algorithm and tumor infiltrating immune cell subsets.

**Figure S3** The expression of the TLR4 gene in KIRC, TGCT, UCEC, SKCM and STAD is correlated with CD4+ T cell, CD8+ T cell, Macrophage, Neutrophil, Myeloid dendritic cell in the six algorithms of TIMER, XCELL, MCPCOUNTER, CIBERSORT, EPIC and QUANTISEQ Analysis. **(A)** Correlation analysis of the TLR4 gene expression in CD4+ T cells (CD4+ T cells non-regulatory, naive, memory, central memory, effector memory, memory activated and resting, Th1 and Th2). **(B)** Correlation analysis of the TLR4 gene expression in Macrophage M0, M1, M2 and Macrophage/Monocyte. **(C)** Correlation analysis of the TLR4 gene expression in CD8+ T cells (CD8+ T cells naive, memory, central memory, effector memory). **(D)** Correlation analysis of TLR4 gene expression in Neutrophil. **(E)** Correlation analysis of the TLR4 expression in Myeloid dendritic cell (Myeloid dendritic cell activated, resting, Plasmacytoid dendritic cell).

**Figure S4** The analysis of the TLR4 gene mutation in KIRC, TGCT, UCEC, SKCM and STAD. **(A)** The mutation frequency of TLR4 in the corresponding tumor samples: SKCM, UCEC, STAD, KIRC were 13.03%, 7.16%, 6.38%, and 0.54%, respectively. **(B)** TLR4 gene somatic mutations in KIRC, SKCM, STAD, TGCT and UCEC are mainly Diploid/Normal, Arm-level Deletion, Arm-level Gain, and High Amplication rarely.

**Figure S5** The relationship between TLR4 gene expression in KIRC, TGCT, UCEC, SKCM and STAD with age, gender, race, and stage, the data from the Cancer Genome Atlas (TCGA) database(n=12332). **(A)** The expression of the TLR4 gene is correlated with the age of KIRC. **(B)** The expression of the TLR4 gene is correlated with the gender of TCGA, KIRC, SKCM and UCEC. **(C)** The expression of the TLR4 gene is correlated with the race of TGCT, KIRC, SKCM and UCEC. **(D)** The expression of the TLR4 is correlated with the stage of KIRC and UCEC.

**Figure S6** The expression of the TLR4 gene in KIRC, TGCT, UCEC, SKCM and STAD tumor stages. **(A)** The expression of the TLR4 in stage I of KIRC was higher than that of stage II, stage

III and stage IV, and the difference was statistically significant at p=0.00105. **(B)** The expression of the TLR4 in stage II stage of TGCT was higher than that in stage III stage. P>0.05, the difference was not statistically significant. **(C)** The expression of the TLR4 in stage I of UCEC was higher than that of stage II, III and IV, P>0.05, the difference was not statistically significant. **(D)** The expression of the TLR4 in stage I of SKCM was higher than that of stage II, stage III and stage IV, and the difference of p<0.05 was statistically significant. **(E)** The expression of the TLR4 in stage IV stage of STAD is higher than stage I stage, stage II and stage III, and the difference of p<0.05 is statistically significant.

**Summarize of the Figure S1-6.**

To make the results more convincing, we conducted more in-depth research on tumor samples. The expression of TLR4 gene in KIRC, TGCT, UCEC, SKCM and STAD has different degrees of positive and negative correlation with tumor infiltrating immune cell subsets in MCPCOUNTER, CIBERSORT, EPIC, QUANTISEQ, TIMER and XCELL algorithms (Figure S1-2). The expression of TLR4 gene in KIRC, TGCT, UCEC, SKCM and STAD is different in the six algorithms of TIMER, xCell, MCP-counter, CIBERSORT, EPIC and QUANTISEQ from CD4+ T cells, CD8+ T cells, Macrophage, Neutrophil, Myeloid dendritic cells the degree of correlation (positive correlation or negative correlation) (Figure S3). In addition to TGCT among the 5 tumors, the TLR4 gene has varying degrees of mutations in the corresponding tumor samples, the TLR4 gene somatic mutations in KIRC, SKCM, STAD, TGCT and UCEC are mainly Diploid/Normal, Arm-level Deletion, Arm-level Gain, and High Amplication rarely (Figure S4). The expression of TLR4 gene is correlated with gender and race of TGCT, KIRC, SKCM and UCEC. Moreover, the expression of TLR4 gene is correlated with the age of KIRC. In addition, the expression of TLR4 gene is correlated with the tumor stages of KIRC and UCEC. (Figure S5). Except for TGCT and UCEC, the expression of TLR4 gene in KIRC, SKCM and STAD tumor stages is consistent with the results of previous studies (Figure S6). Further analysis showed that the TLR4 gene is closely related to tumor infiltrating immune cells. Our research provides a potential target for the development of more immunotherapies and the validation of animal models in the future.
